# Distinguishing Free Cytosolic and Vesicle-Confined Nanoparticle Motion by Fluorescence Correlation Spectroscopy

**DOI:** 10.1021/acs.jpcb.6c05465

**Published:** 2026-09-25

**Authors:** Karina Kwapiszewska, Grzegorz Bubak, Krzysztof Bielec, Aneta Magiera, Alicja Zgorzelska, Robert Hołyst

**Affiliations:** † Institute of Physical Chemistry, 119463 Polish Academy of Sciences, Kasprzaka 44/52, 01-224 Warsaw, Poland; ‡ Department of Chemistry, University of Zurich, Winterthurerstrasse 190, CH-8057 Zurich, Switzerland

## Abstract

Understanding whether internalized nanoparticles remain confined within intracellular vesicles or are released into the cytosol is essential for evaluating their biological activity and potential use as delivery systems. However, this distinction is often difficult to establish by using fluorescence imaging alone, particularly at nanomolar probe concentrations. Here, we propose fluorescence correlation spectroscopy (FCS) combined with length-scale-dependent viscosity analysis and appropriate diffusion models as an approach for assessing nanoparticle uptake and intracellular localization. The method was evaluated in HeLa cells using fluorescent polystyrene nanoparticles, cell-penetrating peptide-coated nanoparticles, dextran of two molecular weights, and receptor-internalized transferrin. Cytosolic probes were identified by autocorrelation curves that fit an anomalous diffusion model and by diffusion coefficients consistent with those predicted by the cytoplasmic length scale-dependent viscosity profile. In contrast, vesicle-associated probes produced characteristic two-step autocorrelation curves that were modeled as the sum of probe diffusion within a confined space, vesicle translation, and directed intracellular transport. Bare nanoparticles, pinocytosed dextrans, and transferrin displayed predominantly vesicle-associated mobility, whereas microinjected dextrans and cell-penetrating-peptide-associated nanoparticles exhibited diffusion in the cytosol. Our results demonstrate that FCS can complement fluorescence imaging by detecting cytosolic probe populations and distinguishing between cytosolic release and vesicular retention.

## Graphical abstract

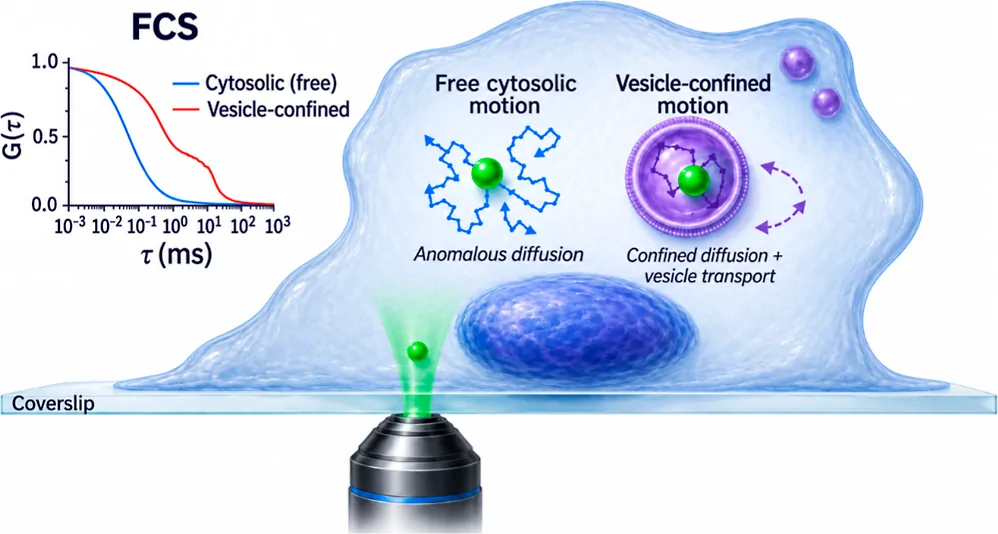

## Introduction

Understanding the mechanisms by which human cells take up nanomaterials is crucial for researchers developing new functional and therapeutic nanomaterials,^1^ as well as for those studying nanomaterial pollution.^2^ Nanomaterials are becoming increasingly prevalent in the human environment, ranging from functional nanoparticles incorporated into everyday products^3^ to nanoparticles engineered as drug nanocarriers.^4,5^ At the same time, the widespread use and fragmentation of plastics contribute to the presence of plastic nanoparticles (nanoplastics) in the environment.^6^ These developments have increased awareness that nanomaterials of diverse origins should be examined for their interactions with the human body. This, in turn, provides the rationale for a broad range of experimental approaches in nanoparticle toxicity research.^7^

A large proportion of studies addressing the biological activity of nanoparticles (NPs) rely on relatively simple cytotoxicity assays,^3^ whereas whole-organism responses to NP exposure are investigated less frequently.^8^ Although cytotoxicity assays provide an initial overview of the potential hazards associated with a given nanomaterial, they usually do not explain the mechanisms underlying nanoparticle-induced effects.^3^ Moreover, potential NP accumulation, intracellular persistence, and delayed toxicity are often insufficiently addressed in standard experimental procedures.^9^ To meet these challenges, techniques that enable monitoring and quantification of the fate of NPs in living cells are needed.

The cellular uptake is a key step determining the potential biological activity of nanoparticles (NPs).^10^ During this process, four parameters may be particularly important: (1) the mechanism of cellular uptake; (2) the number of particles introduced into or associated with the cell, here termed particle-to-cell partitioning or uptake efficiency; (3) accumulation in specific cellular compartments; and (4) the kinetics of NP elimination from the cell.^11,12^ Investigation of all four parameters is required to understand the mechanisms underlying the biological activity of NPs. The techniques most commonly used to address these questions are fluorescence microscopy and flow cytometry.^13^ While flow cytometry enables quantitative analysis of overall NP uptake across a cell population, imaging techniques provide spatial information and mostly qualitative or semiquantitative insight into uptake mechanisms, intracellular localization, and nanoparticle fate.^14^

Here, we discuss the application of quantitative microscopy-based methods for evaluating the NP uptake. Fluorescence correlation spectroscopy (FCS), raster image correlation spectroscopy (RICS), and single-particle tracking (SPT) have previously been used to monitor nanoparticles in living cells.^15–17^ Most of these studies provide information on the presence, localization, and mobility of NPs;^18,19^ however, direct biological interpretation of the measured dynamic parameters remains challenging. This limitation is partly due to the difficulty of linking diffusion coefficients measured in the complex intracellular environment with the actual size, aggregation state, or interactions of the tracked particles.^20^ Moreover, distinguishing whether a particle has been released into the cytosol or remains confined within an intracellular vesicle usually requires more complex or complementary experimental approaches.^21–23^ To overcome this challenge, we propose using FCS, combined with the length-scale-dependent viscosity model [length scale dependent viscosity (LSDV) model],^24,25^ to assess the cellular uptake and cytosolic release of the nanoparticles under study. We use the LSDV model to identify probes of defined sizes that diffuse freely in the cytosol of the cells under investigation. In addition, we established a model to fit FCS data obtained from probes that remain confined within intracellular vesicles. Thus, a single FCS measurement can provide information on the presence, intracellular fate, and quantity of nanoparticles in cells. We demonstrate this approach in HeLa cells, whose intracellular viscosity has been quantitatively characterized in our previous studies.^25,26^ Cells were incubated with various nanoparticles, including dextrans and polystyrene nanobeads, under different uptake conditions: spontaneous uptake, microinjection, and delivery via cell-penetrating peptides (CPPs). FCS curves were acquired and fitted using either an anomalous diffusion model or a confined diffusion model to distinguish probes released into the cytosol from those retained within vesicles (Figure 1).

**Figure 1:**
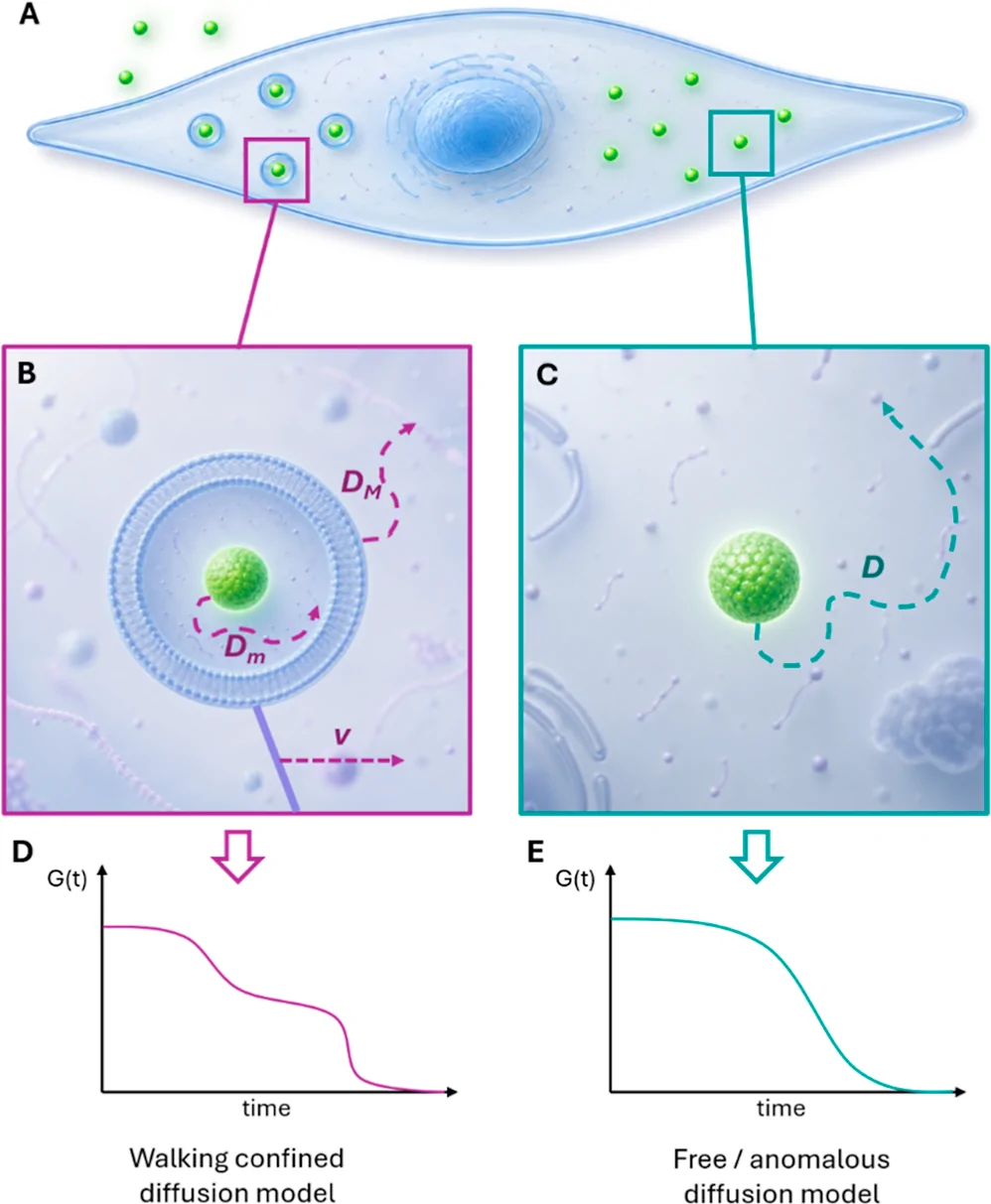
Intracellular nanoparticle uptake scenarios and their consequences for mobility patterns. (A) Nanoparticles are primarily uptaken via endocytosis pathways and very often remain enclosed in vesicles. For certain purposes, the uptake may be engineered to release particles directly into the cytosol. (B) Nanoparticles in vesicles undergo three independent motions: self-diffusion in the confined vesicle space, *D*_m_, self-diffusion of the whole vesicle, *D*_M_, and (very often) active motion led by motor proteins, *v*. (C) Nanoparticles in cytosol exhibit self-diffusion, *D* (up to size constraint of 50 nm).^25^ When measured with FCS, different shapes of the autocorrelation function reflect two scenarios: (D) walking-confined diffusion with a velocity term (eq 7), or (E) the general form of the autocorrelation function (eq 3), with parameters that depend on the nanoparticle type.^20^

## Theory

### Cellular Uptake of Nanoparticles

Nanoparticles enter cells predominantly through endocytic pathways, in which the plasma membrane surrounds extracellular material and internalizes it in vesicular structures.^1,10^ Several types of endocytosis may contribute to the nanoparticle uptake, including clathrin-mediated endocytosis, caveolin-mediated endocytosis, macropinocytosis, and phagocytosis.^27^ The dominant uptake mechanism depends on both the cell type and the physicochemical properties of the nanoparticles, including their size, shape, surface charge, surface chemistry, stiffness, and composition of the biomolecular corona.^1,10^

Clathrin-mediated endocytosis is frequently involved in the uptake of smaller or ligand-functionalized nanoparticles and is associated with the formation of clathrin-coated pits that pinch off from the plasma membrane.^10,27^ Caveolin-mediated endocytosis occurs through caveolae, flask-shaped membrane invaginations enriched in caveolin and cholesterol.^10,27^ Macropinocytosis enables the internalization of nanoparticles into larger, less-structured vesicles formed by actin-driven membrane ruffling.^1,27^ Phagocytosis, which is most prominent in specialized immune cells, is responsible for the uptake of larger particles and particulate material.^1,27^ The efficiency of nanoparticle internalization may depend strongly on the particle size; for some receptor-mediated uptake systems, particles with diameters around 40–50 nm have been reported to show particularly efficient cellular uptake.^28,29^

### Length-Scale Dependent Viscosity

The cytoplasm cannot be treated as a simple, homogeneous liquid. It is a crowded and structurally complex medium in which the mobility of nanoobjects depends not only on temperature and bulk viscosity but also on the size of the diffusing object and on the characteristic length scales of the intracellular environment.^24,25^ Therefore, nanoparticles, proteins, polymers, and small-molecule probes may exhibit different effective viscosities even within the same cytosol. This phenomenon is described by the LSDV model. In this framework, the effective viscosity experienced by a probe is dependent on its hydrodynamic radius. Small probes can diffuse through the spaces between cytoplasmic obstacles and therefore experience relatively low viscosity, close to that of the local solvent-like environment. Larger probes interact with larger cytoplasmic structures and experience a higher effective viscosity. As a result, the apparent viscosity increases with the probe size and approaches the macroscopic viscosity at larger length scales.^24,25^

Experimentally, the LSDV profile of a cell can be obtained by measuring the diffusion coefficients of fluorescent probes of known hydrodynamic radii. In FCS-based measurements, the diffusion coefficient measured in the cytoplasm is compared with the diffusion coefficient of the same probe in a reference solution. Following the Stokes–Sutherland–Einstein relation (Supporting Information, S1), this comparison allows the relative effective viscosity experienced by the probe to be estimated as

1 $$\frac{\eta_{eff}}{\eta_{0}}=\frac{D_{0}}{D}$$

where *D* is the diffusion coefficient measured in the cytoplasm, *D*_0_ is the diffusion coefficient in the reference solution, η_eff_ is the effective viscosity experienced by the probe, and η_0_ is the viscosity of the reference solution.^25^ The LSDV model introduces characteristic length scales describing the intracellular environment, including the effective distance between cytoplasmic obstacles and the size of the major crowders, and their influence on the effective viscosity, as presented in eq 2

2 $$\eta_{eff}=\eta_{0}A\;\exp\left[{\left(\frac{\xi^{2}}{{R_{H}}^{2}}+\frac{\xi^{2}}{{r_{p}}^{2}}\right)}^{-a/2}\right]$$

where *A* is a pre-exponential factor of the order of 1, ξ and *R*_H_ are length scales characteristic of a given system, and *a* is an exponent of the order of unity.^24^ *R*_H_ can be interpreted as the hydrodynamic radius of the main crowders, while ξ denotes an effective intercrowder gap that includes a weak-interaction factor.^25^ These parameters enable the relationship between a given probe’s mobility and the physical structure of the cytoplasm to be established. Measurements performed on probes ranging from small molecules and proteins to polymeric tracers and nanoparticles showed that cytoplasmic viscosity is strongly length-scale-dependent in human cells.^25^ In particular, nanoprobes with diameters from 1 to 150 nm experience different effective viscosities in the cytoplasm, and this dependence is largely conserved across several human cell lines.^25^

This concept is especially important for interpreting FCS data in nano-object uptake experiments.^23,30^ A diffusion coefficient measured inside a cell cannot be directly translated into particle size using simple diffusion models developed for homogeneous liquids. The same diffusion coefficient may reflect different situations, including free cytosolic diffusion, transient interactions with intracellular components, probe polydispersity, or confinement within vesicular structures.^23,30,31^ Thus, a comparison of the measured mobility with the LSDV profile of the cytoplasm provides a reference for assessing whether a nanoparticle-sized object behaves as a freely diffusing cytosolic probe of a given size.

In this work, the LSDV model is used as a reference framework for FCS measurements of nanoparticle uptake and the intracellular fate. Probes that follow the expected LSDV profile for the cytosol are interpreted as being freely diffusing in the cytoplasmic environment. In contrast, probes whose FCS curves indicate strongly hindered or confined motion are interpreted as remaining within intracellular vesicles or other restricted compartments. Previous measurements in HeLa cells showed that the cytoplasmic nanoviscosity remains relatively stable during the cell cycle, supporting the use of this cell line as a reproducible model for FCS-based analysis of intracellular diffusion.^32^

### Model for Diffusion in Cytosol

The FCS autocorrelation function can be generally expressed as (eq 3)­

3 $$G\left(\tau \right)=\frac{1}{N}\sum_{i=1}^{n}A_{i}\frac{1}{1+{\left(\frac{\tau }{\tau_{D,i}}\right)}^{\alpha }}\frac{1}{\sqrt{1+\frac{1}{\kappa^{2}}{\left(\frac{\tau }{\tau_{D,i}}\right)}^{\alpha }}}$$

where *N* is the average number of fluorescent probes inside the focal volume, τ_D,*i*_ is the time of diffusion of an average probe across the focal volume, *A*_*i*_ corresponds to the amplitude of the *i*-th component, and κ is the aspect ratio of the focal volume (derived during calibration).^20^ When measuring the diffusion of a single, monodisperse species in a Newtonian fluid (e.g., water), *n* = 1, *A* = 1, and α = 1, eq 3 simplifies to eq 4.

4 $$G\left(\tau \right)=\frac{1}{N}\frac{1}{1+\frac{\tau }{\tau_{D}}}\frac{1}{\sqrt{1+\frac{1}{\kappa^{2}}\frac{\tau }{\tau_{D}}}}$$

In the living cell cytoplasm, the anomalous diffusion model (α ≠ 1) better describes the diffusion of nanomaterials.^20^ It is due to the length-scale dependence of diffusion hindrance. Real nanomaterial samples contain particles of varied sizes, although the dispersity, by definition, may be ∼1.^33^ When diffusing in the complex liquid-like cytoplasm, particles of different sizes experience different diffusion hindrance (increasing with particle size; see chapter Length-Scale Dependent Viscosity), which broadens the distribution of diffusion times. As a consequence, apparent diffusion is a subdiffusion type, meaning that α < 1, as more broadly explained in ref 20 (eq 5).^20^

5 $$G\left(\tau \right)=\frac{1}{N}\frac{1}{1+{\left(\frac{\tau }{\tau_{D}}\right)}^{\alpha }}\frac{1}{\sqrt{1+\frac{1}{\kappa^{2}}{\left(\frac{\tau }{\tau_{D}}\right)}^{\alpha }}}$$

Equation 5 describes diffusion of polymers and nanoparticles released into the cytosol of mammalian cells.^20,25,26^

### Model for Diffusion in Vesicles

When the nanoprobe is enclosed in a vesicle, two motions are combined: translational diffusion inside the vesicle and movement of the vesicle itself. Previously, Ochab-Marcinek et al.^34^ introduced a model for walking confined diffusion, describing diffusion inside a vesicle also moving by diffusion (eq 6)­

6 $$G\left(\tau \right)={\left(1+\frac{2}{3}\frac{\left[6D_{M}\tau +\frac{6}{5}{R_{v}}^{2}\left(1-e^{-5D_{m}\tau /{R_{v}}^{2}}\right)\right]}{{\omega_{xy}}^{2}}\right)}^{-1}\times {\left(1+\frac{2}{3}\frac{\left[6D_{M}\tau +\frac{6}{5}{R_{v}}^{2}\left(1-e^{-5D_{m}\tau /{R_{v}}^{2}}\right)\right]}{\kappa^{2}{\omega_{xy}}^{2}}\right)}^{-1/2}$$

where *D*_M_ was defined as the diffusion coefficient of the confined space—the vesicle in our case, *D*_m_ is the diffusion coefficient of the nanoprobe inside the vesicle, *R*_v_ is the radius of the vesicle, ω_*xy*_ is the radius of the waistband of the focal volume, and κ is the focal volume aspect ratio. Equation 6 describes a situation where the vesicle undergoes free diffusion in the cytoplasm, which is not true. Vesicles in eukaryotic cells usually undergo active intracellular transport mediated by molecular motors, including kinesins, dyneins, and myosins.^35^ Thus, we added a term, describing active transport of the vesicle, as presented in eq 7

7 $$G\left(t\right)={\left(1+\frac{2}{3}\frac{\left[6D_{M}t+\frac{6}{5}{R_{v}}^{2}\left(1-e^{-5D_{m}t/{R_{v}}^{2}}\right)\right]}{{\omega_{xy}}^{2}}\right)}^{-1}\times {\left(1+\frac{2}{3}\frac{\left[6D_{M}t+\frac{6}{5}{R_{v}}^{2}\left(1-e^{-5D_{m}t/{R_{v}}^{2}}\right)\right]}{\kappa^{2}{\omega_{xy}}^{2}}\right)}^{-1/2}\times e^{-{\left(\frac{tv}{\omega_{xy}}\right)}^{2}}$$

where *v* is the linear velocity of a molecular motor dragging the vesicle.

## Methods

### Reagents

Carboxyl-modified polystyrene nanobeads with a probe radius (rp) of 14 nm, as determined by the manufacturer, were purchased from ThermoFischer (FluoSpheres). NP was labeled with a dye whose emission is observed in the yellow-green (YG) region of the spectrum (510 nm). Provided aqueous suspension of nanoparticles was diluted to the desired concentration in different buffers. The sample concentration was controlled using FCS. Fluorescent TRITC-dextran conjugates with molecular masses of 20,000 Da (product number 73766) and 155,000 Da (product number T1287) were ordered from Sigma-Aldrich (Saint Louis, Missouri, United States). Transferrin from Human Serum, Alexa Fluor 647 Conjugate, was purchased from Thermo Fisher.

CPPs: nona-arginine (Arg)_9_ peptide in non- and TAMRA-labeled forms were purchased from Eurogentec. The amino acid composition of peptides means that at pH 7.4, their overall positive charge is approximately +8.7. Prepared 500 μM aliquots were stored in −20 °C. The electrostatic conjugation with NP was performed by the addition of small amounts of CPPs to vortexed suspension up to the final ratio unless stated otherwise.

### Cell Culture

HeLa cells came from ATCC (Manassas, USA). They were routinely cultured as monolayer in the Dulbecco’s modified Eagle’s medium with 1 g/L glucose (Institute of Immunology and Experimental Technology, Wrocław, Poland), supplemented with 10% v/v fetal bovine serum (FBS, Gibco), l-glutamine 1% v/v (Sigma-Aldrich), and the antibiotics: streptomycin [10 mg/mL] and penicillin [10 000 U/ml] 1% v/v (Sigma-Aldrich). Culture was performed in an incubator at 37 °C with a 5% CO_2_ concentration. Cells were maintained in a logarithmic growth phase. 0.25% Trypsin–EDTA solution (Sigma-Aldrich) was used to detach cells from the surface.

Cells were grown on an 8-chamber cover glass slide (Cellvis) for FCS measurements and confocal imaging. Experiments were performed 24 h after cell seeding. Microinjection was performed using the Femtojet system (Eppendorf) with glass capillaries of <1 μm diameter, prepared with a micropipette puller (P-1000, Sutter Instrument). The concentrations of injected solutions were ∼20 μM (in PBS). Imaging and FCS measurements were performed 1 h after microinjection.

### Microscope Setup

Fluorescence-based measurements: FCS, brightness analysis, and the photon counting method were performed on the confocal microscope system, which is based on a Nikon C1. The microscope is equipped with a 60× objective (Nikon PlanApo, NA = 1.2) and a PicoQuant LSM system with two single-photon avalanche diode detectors, controlled in parallel by a PicoHarp 300 TCSPC. As the excitation sources, we used 481, 561, and 647 nm pulsed diode lasers. For time-dependent laser multicolor control, we use a modular Sepia II PDL 828 driver. The whole microscope system is controlled by SymPhoTime software. The temperature during measurements was maintained at the desired values (25 °C for buffer experiments and 36 °C for cell experiments) within an insulated external box manufactured by OkoLab.

### Data Acquisition and Analysis

FCS autocorrelation curves (ACCs) were calculated by using SymphoTime software with fluorescence lifetime-based background correction. Next, ACCs were transferred to FcsIT open-source software (https://github.com/TKmist/FcsIT)^36^ to fit the appropriate mathematical model to the FCS curves.

## Results

### In Vitro Characterization of the Probes

Hydrodynamic properties of nanoparticles (NPs), fluorescently labeled dextrans, proteins, and CPPs were characterized. We have adjusted the laser power for each compound characteristic used in the measurements to avoid photobleaching and maintain an appropriate signal-to-noise ratio. The excitation laser power for fluorescent NPs was set to 1 μW (481 nm). TAMRA-labeled CPPs were excited by a green laser (561 nm) and showed a linear response up to 20 μW. Similarly, TRITC-labeled dextrans were excited by a 561 nm laser at 5 μW, and transferrin-AF647 was measured using a 647 nm laser. As a next step, we performed a series of FCS measurements to obtain the diffusion coefficients of NPs and dextrans in water, and transferrin and CPPs in PBS buffer. These in vitro experiments were performed at 25 °C. The determined diffusion coefficient can be used to calculate the hydrodynamic radius (*r*_h_) using the Einstein–Stokes equation, as explained in the Supporting Information (S1). Based on FCS measurements, we calculated the *r*_h_ of the probes and listed them in Table 1.

**Table 1:** In Vitro Characterization of the Nanoobjects: FCS in Water or PBS, 25 °C

probe	buffer	measured diffusion coefficient [μm^2^/s]	hydrodynamic radius, *r*_p_ [nm]	diffusion coefficient expected in cytoplasm of HeLa cells [μm<sup>^2^/s]
nanoparticles (NP)	water	11	22	1.5
NP–CPP complex	PBS^a^	8.1	30	0.94
TRITC-dextran 20 kDa	water	65	3.8	28
TRITC-dextran 155 kDa	water	29	8.6	4.9
transferrin AF647	PBS^a^	57	4.3	

a Phosphate-buffered saline, ionic strength 0.164 M, pH = 7.4.

For the nanoparticle–CPP conjugation, nanoparticle surface saturation was determined by incubating a constant concentration of nanoparticles with increasing CPP concentrations and monitoring changes in the diffusion coefficient, indicating saturation at approximately 800 CPP molecules per nanoparticle (see Supporting Information S2).

### Cellular Uptake of Bare and CPP-Coated NPs

Following the determination of the diffusion coefficients of unconjugated NPs and NP–CPP conjugates, their intracellular localization and mobility were investigated in HeLa cells. The experiment aimed to determine how the presence of CPPs affects the cellular distribution, accumulation pattern, and intracellular mobility of the nanoparticles.

Unconjugated NPs and NP–CPP conjugates were added to the cell culture medium and incubated with the cells overnight to allow for the cellular uptake. Stock solutions were prepared at a concentration of 200 nM. The NP–CPP conjugates were prepared at a surface ratio of (*R* = 0.3), while unconjugated NPs were used as the corresponding control. A 4 μL volume of each stock solution was added to 200 μL of cell culture medium. Cell confluence at the time of probe addition was approximately 40%. According to the LSDV model, the diffusion coefficients of unconjugated NPs and NP–CPP conjugates in the cytosol were expected to decrease to approximately 1.5 and 0.94 μm^2^/s, respectively (Table 1). The experimental results are presented in Figure 2.

**Figure 2:**
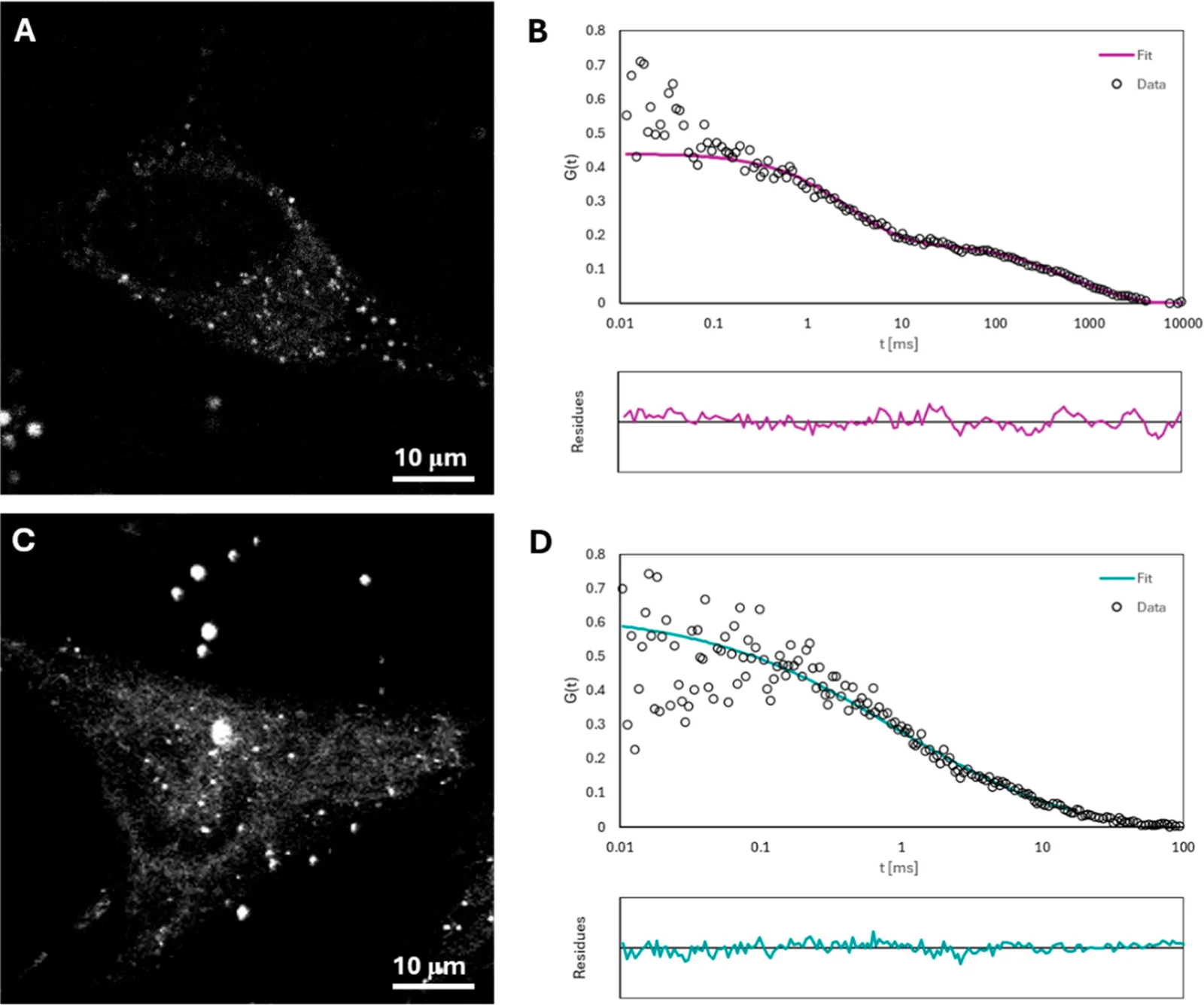
Uptake of nanoparticles into HeLa cells. (A) Cells incubated with bare nanoparticles exhibited fluorescent spots in the cytoplasmic area. (B) FCS performed in the (A) sample yielded a characteristic, two-step autocorrelation curve, which was perfectly fitted with eq 7. (C) Intracellular release of CPP-nanoparticles could not be unambiguously determined from imaging. For this sample (D), FCS in the cytosol yielded data that fitted a single-component anomalous diffusion model (eq 5), confirming cytosolic release of the probe. Scale bars correspond to 10 μm.

Unconjugated NPs did not show pronounced aggregation on the glass surface of the culture vessels. Following the cellular uptake, however, the nanoparticles tended to accumulate in specific intracellular regions, as indicated by the localized high-intensity fluorescence spots. The FCS performed in the cytoplasmic area of NP-containing cells exhibited a clear two-step shape (Figure 2B). This indicated enclosure within intracellular vesicles, which was further confirmed by fitting to diffusion in a moving confined space (eq 7). Perfect fitting of the model to the experimental data suggests an agreement with the predicted physical model.

A different mobility pattern was observed for the NP–CPP conjugates. Consistent with the *in vitro* measurements, a fraction of the conjugates formed aggregates on the glass surface of the culture vessel. Nevertheless, the conjugates detected within the cells showed a slightly more homogeneous intracellular distribution than the unconjugated nanoparticles; however, a clear distinction in the pattern could not be made based on imaging alone (Figure 2A,C). To explore differences in mobility, FCS autocorrelation curves were acquired in the cytoplasmic area. The curves were fitted with a single-component anomalous diffusion model (eq 5)—a model characteristic for nanoparticles in cytosol.^20^ The measured diffusion coefficient was 1.3 ± 0.8 μm^2^/s, which was in good accordance with the predicted values (Table 1). However, the measurement accuracy was insufficient to unequivocally determine whether the nanoparticles remained complexed with CPPs within the cells or had dissociated.

### Microinjection vs Pinocytosis of Dextrans

To validate the proposed FCS fitting strategy, TRITC-labeled dextrans were examined under two experimental conditions for which the expected intracellular localization and mobility profiles had previously been established.^20,37^ Direct microinjection was used to introduce dextrans into the cytoplasm,^20^ whereas incubation with dextrans in the presence of 1 M sucrose resulted in their uptake and retention within pinocytic vesicles.^37^ These two delivery routes were therefore expected to produce clearly distinguishable FCS autocorrelation curves.

Following microinjection, both 20 and 155 kDa TRITC-dextrans were distributed relatively evenly throughout the cytoplasm. However, this was not clearly visible in the images as the nanomolar probe concentrations suitable for FCS were close to the detection limit of fluorescence imaging (Figure 3A,B). The corresponding FCS autocorrelation curves were successfully fitted using the anomalous diffusion model described by eq 5 (Figure 3E,F). Apparent anomalous diffusion of dextrans in cells is a direct effect of the probe’s polydispersity and the complexity of the cytoplasm, which is consistent with our previous work.^20,25^

**Figure 3:**
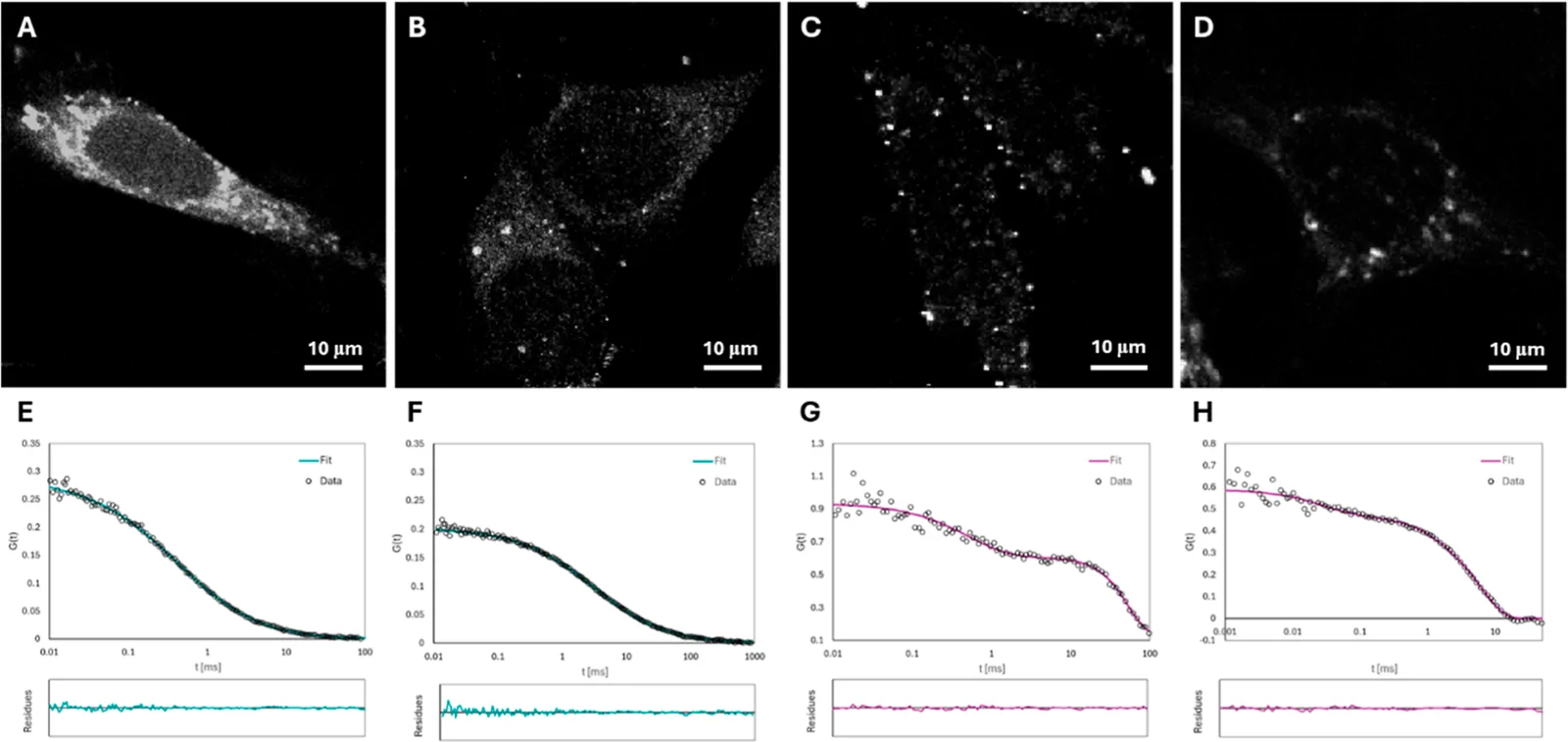
Determination of intracellular localization of TRITC-dextrans. TRITC-dextrans: (A) 20 and (B) 155 kDa, were microinjected directly into the cytoplasm of HeLa cells. These samples yielded FCS curves fitted with the anomalous diffusion model (eq 5), for both (E) 20 and (F) 155 kDa dextrans. In the other experiment, cells were incubated with (C) 20 and (D) 155 kDa TRITC-dextrans in the presence of 1 M sucrose. This scenario resulted in numerous pinocytic vesicles containing fluorescent probes, which resulted in FCS curves fitted with eq 7 ((G) 20, (H) 155 kDa). Scale bars correspond to 10 μm.

In contrast, the spontaneous uptake upon incubation with 20 kDa or 155 kDa TRITC-dextrans in the presence of sucrose resulted in a punctate fluorescence pattern, with numerous fluorescent vesicles visible inside the cells (Figure 3C,D). Under these conditions, the measured fluctuations reflected the mobility of dextrans confined within and transported along with the intracellular vesicular structures. The resulting autocorrelation curves exhibited the expected complex mobility profile and were successfully fitted using eq 7 (Figure 3G,H). For both dextran molecular weights, cytoplasmic delivery yielded autocorrelation curves fitted with the anomalous diffusion model, whereas the vesicular uptake yielded curves fitted with eq 7.

### Transferrin Uptake

To further examine the model-selection strategy in a receptor-mediated endocytic system, the intracellular mobility of Alexa Fluor 647-labeled transferrin was analyzed. Transferrin is internalized together with the transferrin receptor through clathrin-mediated endocytosis. Following endosomal acidification, iron is released, whereas apotransferrin remains associated with the receptor during intracellular recycling and dissociates upon the complex’s return to the cell surface.^38^ Consequently, the intracellular movement of fluorescent transferrin was expected to be coupled predominantly to the movement of transferrin receptor-containing vesicles, with only a limited contribution from independent motion within the vesicular lumen.

Incubation of HeLa cells with AF647-transferrin resulted in a punctate intracellular fluorescence pattern, corresponding to numerous transferrin-containing vesicular structures distributed throughout the cytoplasm (Figure 4A). FCS measurements performed in cytoplasmic regions yielded autocorrelation curves that were fitted using eq 7 (Figure 4B).

**Figure 4:**
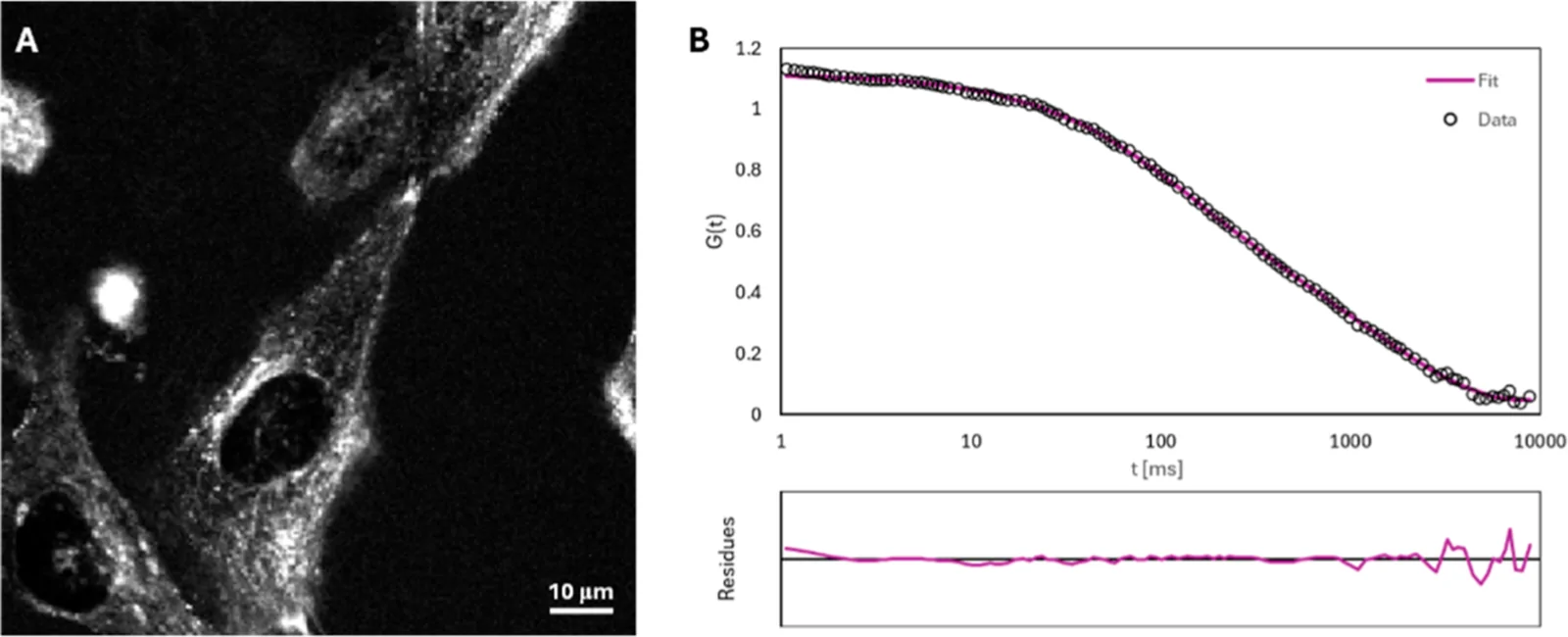
Transferrin uptake into HeLa cells. (A) AF647 transferrin was incubated with HeLa cells, resulting in the visualization of intracellular vesicles. Scale bar corresponds to 10 μm. (B) FCS performed in the cytoplasmic area yielded in curves fitted with eq 7.

Thus, the previously applied fitting model for the vesicle-associated mobility of dextrans and nanoparticles was also selected for transferrin internalized via receptor-mediated endocytosis. In contrast to nanoparticles enclosed within the vesicular lumen, transferrin was expected to remain predominantly associated with the vesicle membrane through its interaction with the transferrin receptor, and this was visible in a significantly increased proportion of the second step of the curve (Figure 4B). The transferrin experiment, therefore, provided an additional reference system in which the measured fluorescence fluctuations were expected to reflect primarily vesicle mobility rather than the independent intravesicular motion of the fluorescent probe.

## Discussion

Determining whether an internalized fluorescent probe remains enclosed within membrane-bound compartments or has been released into the cytosol is not straightforward by using fluorescence imaging alone. Vesicle-associated probes are locally concentrated and therefore produce bright punctate signals, whereas a cytosolic fraction is distributed over a much larger volume and may remain weak or undetectable. This problem becomes particularly relevant at the physiologically relevant nanomolar probe concentrations, which may be close to the detection limit of conventional fluorescence imaging. Consequently, the presence of fluorescent puncta provides evidence for vesicular localization, but imaging alone may be insufficient to establish whether a smaller fraction of the probe has reached the cytosol (refer to microscopic images in Figures 2–4).

This distinction is important in the development of intracellular delivery systems. In the case of nucleic acids and nanoparticle-based drug carriers, the cellular uptake does not necessarily imply delivery of the therapeutic cargo to its cytosolic target because only a fraction of the internalized material may escape from endosomes.^21,39^ Dedicated quantitative imaging approaches have shown that endosomal escape may occur through relatively rare, spatially restricted events that are difficult to detect in standard fluorescence images. A similar limitation applies to the internalization of therapeutic antibodies and other proteins.^23^ Fluorescent intracellular structures may indicate uptake and trafficking, but do not, by themselves, establish the cytosolic bioavailability of the antibody. Functional reporters, single-molecule methods, or complementary mobility measurements are therefore required to distinguish endosomal entrapment from cytosolic delivery.^21–23,39^

Our results indicate that the analysis of FCS autocorrelation curves can provide information complementary to fluorescence imaging. Bare nanoparticles, pinocytosed dextrans of both investigated molecular weights, and receptor-internalized transferrin produced autocorrelation curves with a characteristic two-step decay that could be described using eq 7. In contrast, dextrans delivered directly to the cytoplasm by microinjection produced curves described by the anomalous diffusion model in eq 5. A similar cytosolic mobility profile was observed for the CPP-associated nanoparticles. Importantly, the relationship between the delivery route and the selected model was reproduced by using probes with different sizes and physicochemical properties. Thus, the assignment of eq 7 to vesicle-associated mobility was supported not only by the quality of individual fits but also by control experiments in which the expected intracellular localization was known from the delivery procedure.

Equation 7 represents the measured dynamics as a combination of probe diffusion within a confined space, the translational movement of the vesicle, and a directed transport component. This description captured the experimentally observed autocorrelation profiles for vesicle-associated nanoparticles, dextran, and transferrin. The transferrin experiment provides particularly useful reference conditions. Following receptor-mediated endocytosis and endosomal acidification, apotransferrin remains predominantly associated with the transferrin receptor during recycling.^38^ Its independent motion within the vesicular lumen is therefore expected to contribute less to the measured fluctuations than in the case of soluble dextran. The pronounced slow component of the transferrin autocorrelation curve is consequently consistent with fluorescence fluctuations dominated by the movement of the transferrin-containing vesicles. One should be aware that agreement between eq 7 and the autocorrelation curve should be interpreted together with the fitting residuals, fluorescence distribution, delivery conditions, and reference experiments as different transport mechanisms may produce similar FCS profiles.^40^

In principle, eq 7 may also provide quantitative information on vesicular transport. Parameters obtained from fitting may include the apparent vesicle radius, the probe’s diffusion coefficient within the vesicle, and the directed vesicle velocity (Table 2). As shown in Table 2, the vesicles’ radii could have been determined with good accuracy and relatively low error. This reflects conservation of vesicular nanoarchitecture across different experiments. Intravesicular diffusion coefficients, *D*_m_, exhibited greater variability, as expected for confined systems.^40^ Moreover, relatively lower *D*_m_ for dextrans than nanoparticles results from the presence of sucrose in the system. The highest variability and uncertainty were observed for the velocity (*v*) values. However, these parameters should initially be described as apparent or effective values. In particular, the fitted velocity *v* represents the net directed velocity of the vesicle during the measurement rather than the intrinsic stepping velocity of an individual motor protein. Intracellular vesicle transport commonly consists of directed runs interrupted by pauses, reversals, detachment events, and changes between cytoskeletal tracks. Multiple motors may also act simultaneously or sequentially on the same vesicle.^35^ A constant velocity term is therefore a useful approximation of the average directed contribution but it does not reproduce the full complexity of motor-driven transport. We propose treating this as a qualitative term (presence or absence of a velocity term), rather than quantitative.

**Table 2:** Quantitative Analysis of Vesicular Transport in HeLa Cells

probe	*D*_m_ [μm^2^/s]	vesicle radius, *R*_v_ [μm]	directed motion velocity, *v* [μm/s]
TRITC-dextran 20 kDa	8.7 ± 2.7	0.20 ± 0.03	2.4 ± 0.9
TRITC-dextran 155 kDa	2.7 ± 2.6	0.26 ± 0.13	0.39 ± 0.68
nanoparticles	4.7 ± 3.2	0.36 ± 0.11	0.17 ± 0.16
transferrin (AF647)		0.14 ± 0.05	0.04 ± 0.01

A further challenge for model development is to describe intermediate conditions in which part of the probe remains confined within vesicles, while another fraction has been released into the cytosol. The current model assumes predominantly either cytosolic diffusion or predominantly vesicle-associated transport. An intermediate situation is likely to occur during incomplete endosomal escape and may be particularly relevant for delivery systems optimized by using CPPs or membrane-disrupting components. A possible extension could combine the cytosolic and the vesicular correlation functions. However, the fitted amplitudes do not directly represent the molecular fractions in each compartment. A vesicle containing multiple fluorescent probes can be considerably brighter than an individual cytosolic molecule. It may therefore dominate the autocorrelation function, even when it contains only a limited fraction of the total probe population. Quantitative estimation of released and vesicular fractions would require brightness calibration, constraints on the number of probes per vesicle, or complementary photon counting or fluorescence-brightness analysis. Bright aggregates and vesicular events are known to disproportionately affect fluctuation correlation measurements and may require dedicated analysis procedures. Moreover, the number of fitting parameters in such a complex model would make it prone to artifacts and overinterpretation.

Another challenge arises from the single-point character of the current FCS measurements. The confocal observation volume samples only a small and fixed region of the cell. Vesicles enter this volume intermittently, and only a limited number of vesicle passages may occur during an individual acquisition. A few bright events may strongly influence the resulting autocorrelation curve and may not represent the full distribution of vesicle sizes, velocities, directions, or transport states in the cell. Differences in local cytoskeletal organization and in distance from the nucleus or plasma membrane may further contribute to variability. The relatively large dispersion of the fitted vesicular parameters should therefore be interpreted as a combination of biological heterogeneity and limited event statistics rather than solely as fitting uncertainty. To address this challenge, several technical approaches could improve the statistical characterization of vesicular motion. Repeated measurements at multiple positions would provide the most direct extension of the present workflow. Scanning or line-scan FCS can repeatedly sample multiple spatial positions.^41^ Raster image correlation spectroscopy could provide spatial maps of molecular mobility across larger cellular regions,^42^ although highly fluorescent vesicles would require careful separation from the faster-diffusing cytosolic fraction. Single-particle tracking would provide distributions of vesicle velocities, run lengths, pauses, and directional changes rather than an ensemble-averaged velocity. Direct comparisons have shown that particle tracking and temporal image correlation can provide complementary estimates of endosome motility. However, both methods are sensitive to the signal-to-noise ratio, immobile structures, and segmentation quality.^43^ Imaging FCS using fast cameras or detector arrays could additionally increase the number of simultaneously sampled observation volumes while retaining sensitivity to weak cytosolic fluorescence. In practice, the most informative strategy may be to combine multiple techniques. TEM and vesicle–dye colocalization offer information that may complement the dynamics-based FCS approach presented here. Correlative light and transmission electron microscopy (CLEM), combining fluorescence colocalization with vesicle/endosomal markers (e.g., LysoTracker, Rab5/Rab7) with ultrastructural TEM imaging, provides direct structural visualization of individual nanoparticles within endolysosomal compartments versus the cytoplasm.^44,45^ However, FCS reports probe mobility with high sensitivity at nanomolar concentrations, CLEM adds static, high-resolution spatial details: vesicle morphology, membrane integrity, and nanoparticle clustering, which dynamic measurements alone cannot capture.^44–46^ Combining the two would allow the same nanoparticle population to be characterized both dynamically and structurally, providing a more complete picture of vesicle-trapped versus cytoplasmic distribution.

Overall, the present approach should be regarded primarily as a dynamics-based method for distinguishing cytosol-like and vesicle-associated mobility profiles rather than as a universal endosomal escape assay. Its main advantage is the ability to detect weak, nanomolar cytosolic probe populations that may be difficult to identify with conventional imaging. The use of microinjected dextrans, pinocytosed dextrans, and receptor-bound transferrin provides a solid reference system, supporting the proposed model-selection strategy. Still, we acknowledge some sources of uncertainty, including incomplete release, heterogeneous vesicle populations, brightness differences, parameter covariance, and limited single-point statistics. Future development incorporating mixed-population models and spatially resolved correlation or particle-tracking measurements could expand the method toward a more complete, quantitative characterization of intracellular trafficking.

## Conclusions

Fluorescence correlation spectroscopy provides information about the nanoparticle uptake that cannot be obtained reliably from fluorescence intensity or spatial distribution alone. By combining FCS measurements with the length-scale-dependent viscosity profile of the cytoplasm and models that describe anomalous cytosolic diffusion and transport in a moving confined space, we distinguished probes released into the cytosol from those retained within intracellular vesicles. The relationship between the selected diffusion model and intracellular localization was confirmed for nanoparticles, dextran, and transferrin introduced through different delivery pathways.

The proposed approach should primarily be regarded as a dynamics-based method for identifying cytosol-like and vesicle-associated mobility rather than as a fully quantitative assay of endosomal escape. Its main advantage is its sensitivity to weak cytosolic fluorescence at nanomolar concentrations, where conventional imaging may fail to reveal the release fraction. We also postulate further development of the proposed methodology to achieve a more in-depth and quantitative characterization of endosomal release and intracellular trafficking beyond the proposed proof-of-concept approach.

## Supplementary Material



## Notes

Details of the Stokes–Sutherland–Einstein relation used to calculate hydrodynamic radii; details of nanoparticle-(Arg)_9_ peptide conjugation and determination of the CPP-to-NP surface saturation ratio; FCS results for YG-NP electrostatically conjugated with (Arg)_9_ peptide at different surface ratios; and image of aggregated YG-NP-(Arg)_9_ conjugates (*R* = 0.3) at the glass surface (PDF)
