# Supplementary material for: Distinguishing Free Cytosolic and Vesicle-Confined Nanoparticle Motion by Fluorescence Correlation Spectroscopy
Source: J Phys Chem B. 2026 Sep 25;130(40):10477–87. doi: 10.1021/acs.jpcb.6c05465 (PMC13656738; doi:10.1021/acs.jpcb.6c05465)
Supplement: Supplementary file 1 [file jp6c05465_si_001.pdf]

Supporting Information

to

Distinguishing Free Cytosolic and Vesicle-Confined Nanoparticle Motion by Fluorescence Correlation Spectroscopy

Karina Kwapiszewska[a,*], Grzegorz Bubak[a], Krzysztof Bielec[a, b], Aneta Magiera[a], Alicja Zgorzelska[a], Robert Hołyst[a]

[a] *Institute of Physical Chemistry, Polish Academy of Sciences, Kasprzaka 44/52, 01-224 Warsaw, Poland*

[b] *University of Zurich, Department of Chemistry, Winterthurerstrasse 190, CH-8057 Zurich, Switzerland*

* corresponding author: kkwapiszewska@ichf.edu.pl

SI.1 Stokes-Sutherland-Einstein relation

The translational diffusion coefficient of a spherical particle in a liquid can be estimated using the Stokes–Sutherland–Einstein (SSE) relation:

$$D = \frac{k_B T}{6\pi\eta r_p}$$

where D is the translational diffusion coefficient, k_B is the Boltzmann constant, T is the absolute temperature, η is the dynamic viscosity of the solvent, and r_p is the hydrodynamic radius of the diffusing object.

The hydrodynamic radius can therefore be calculated from the experimentally determined diffusion coefficient as:

$$r_p = \frac{k_B T}{6\pi\eta D}$$

The relation assumes that the diffusing object can be approximated as a rigid sphere moving in a continuous Newtonian fluid under low-Reynolds-number conditions, with a no-slip boundary condition at the particle surface. It is most accurate for dilute solutions in which intermolecular interactions, aggregation, and macromolecular crowding effects are negligible.

In this work, the SSE relation was used for FCS-derived data to calculate r_p from measured D. All calculations for *in vitro* data were performed at 25 °C (T = 298.15 K). For aqueous solutions, the dynamic viscosity of water at this temperature was taken as approximately 0.890 mPa·s. The same value was used as an approximation for phosphate-buffered saline.

SI.2 NP-peptide conjugation

Before the *in cellulo* experiments, the CPP-to-NP ratio required to saturate the nanoparticle surface was determined. As the manufacturer does not provide information on the number of carboxyl groups present on the NP surface, the total surface area of a YG-NP was calculated to be 2496 nm². Based on the calculated hydrodynamic radius, the surface area occupied by a single peptide molecule was assumed to be 3.1 nm².

To determine the saturation ratio, the NP concentration was kept constant, while the CPP concentration was varied. The samples were incubated for 2 h at 25 °C. The results are presented in Figure S1.

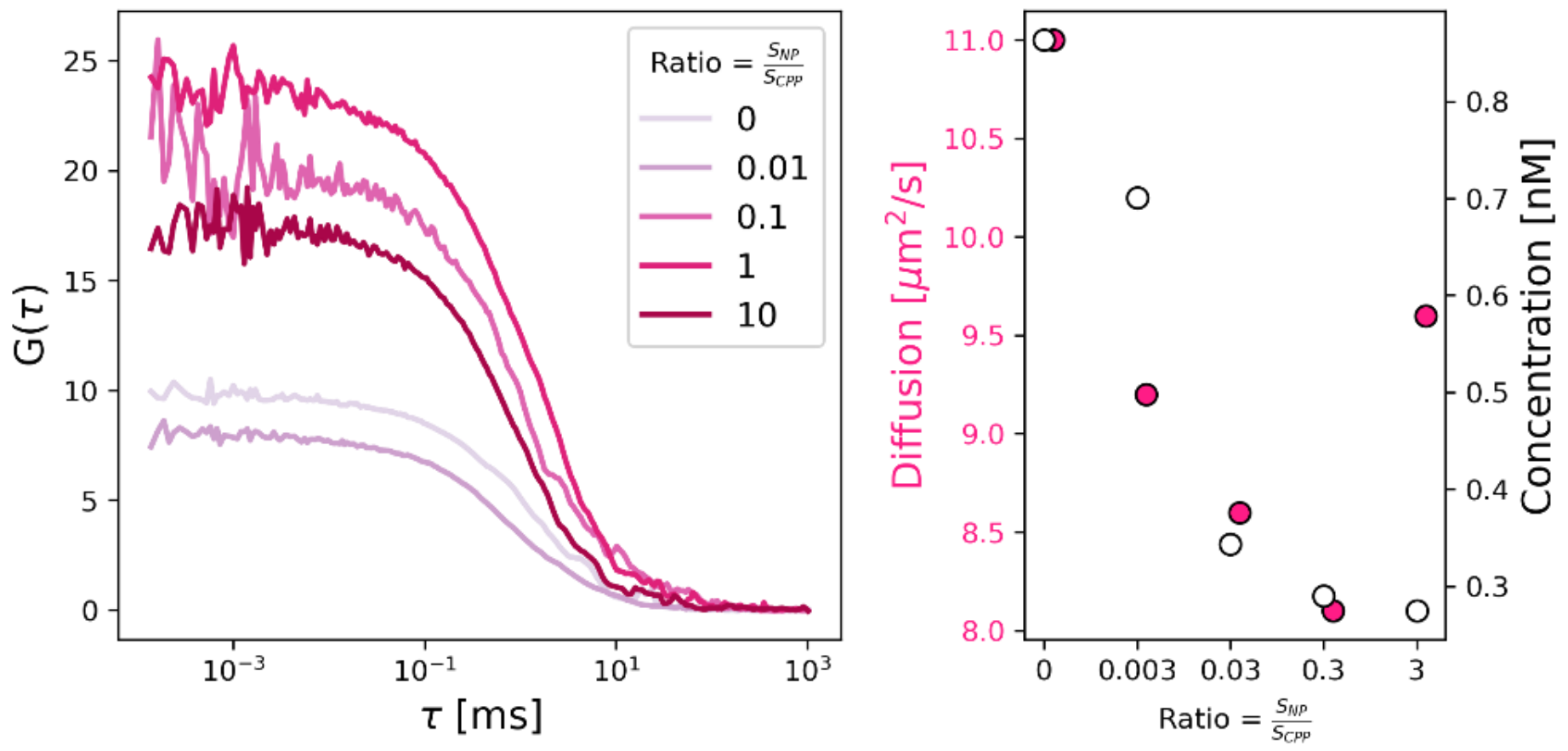

Figure S1 YG-NP were electrostatically conjugated with $(Arg)_9$ peptide in deionized water at different surface ratios.

Above a CPP-to-NP ratio of (R = 0.3), further addition of the peptide did not result in a significant change in the diffusion coefficient, indicating saturation of the NP surface. Based on the surface-area calculations, approximately 800 CPP molecules were estimated to saturate a single NP. For the analysis of electrostatic interactions, it was assumed that one NP contains up to 5600 carboxyl groups, corresponding to approximately 2.26 carboxyl groups per nm².

The experiment was subsequently repeated using the same CPP-to-NP ratios in PBS. Following incubation, no freely diffusing fluorescent probes were detected in the bulk solution. Most fluorescence signals were observed directly above the bottom of the sample container. These observations indicated that the NP–CPP conjugates precipitated and adsorbed onto the glass surface, as shown in Figure S2.

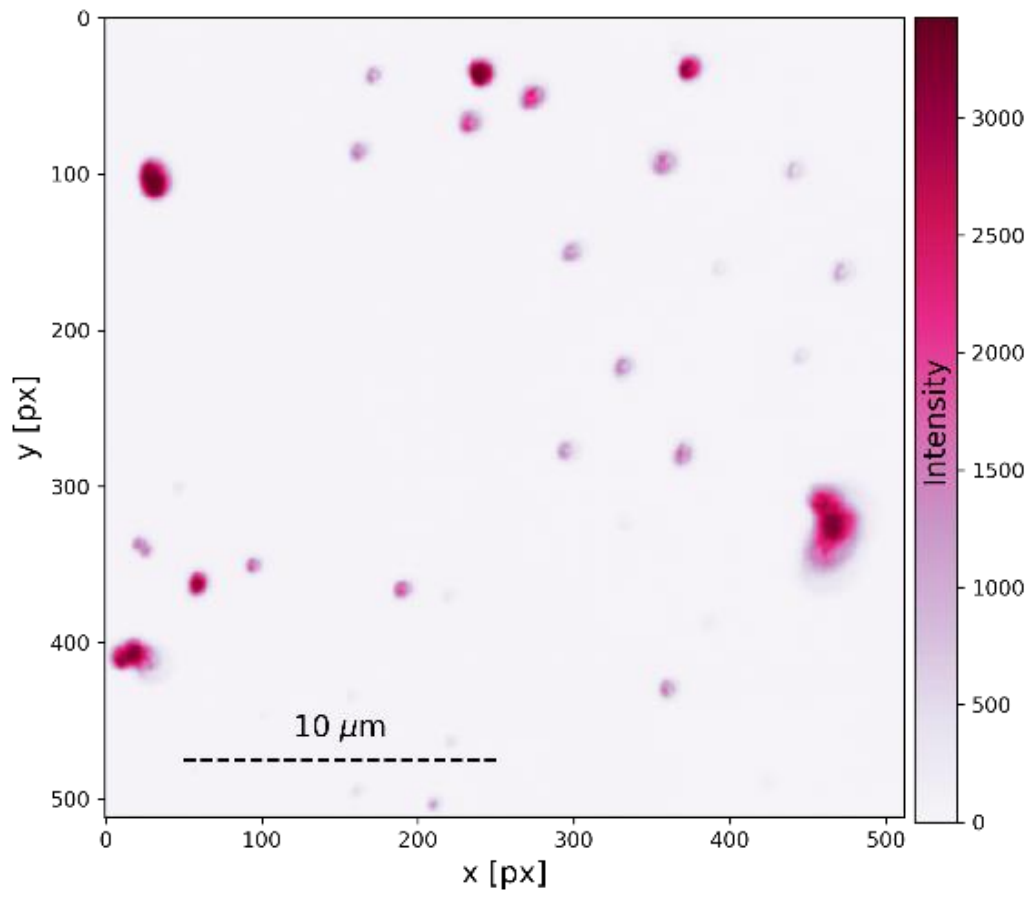

Figure S2. Aggregated YG-NP-$(Arg)_9$ conjugates (R=0.3) at glass surface.
